# Prognostic impact of CXCL16 and CXCR6 in non-small cell lung cancer: combined high CXCL16 expression in tumor stroma and cancer cells yields improved survival

**DOI:** 10.1186/s12885-015-1446-z

**Published:** 2015-05-29

**Authors:** Sigurd M. Hald, Yury Kiselev, Samer Al-Saad, Elin Richardsen, Charles Johannessen, Marte Eilertsen, Thomas K. Kilvaer, Khalid Al-Shibli, Sigve Andersen, Lill-Tove Busund, Roy M. Bremnes, Tom Donnem

**Affiliations:** 1 Department of Clinical Medicine, UiT The Arctic University of Norway, 9037 Tromso, Norway; 2 Department of Oncology, University Hospital of North Norway, Tromso, Norway; 3 Department of Medical Biology, UiT The Arctic University of Norway, Tromso, Norway; 4 Department of Pathology, Nordland Hospital, Bodo, Norway; 5 Department of Clinical Pathology, University Hospital of North Norway, Tromso, Norway; 6 Department of Pharmacy, UiT The Arctic University of Norway, Tromso, Norway

## Abstract

**Background:**

The chemokine CXCL16 and its receptor CXCR6 are expressed by a variety of immune cells and have been shown to influence angiogenesis. The expression of CXCR6 and CXCL16 has been examined in numerous human cancers; however no studies have yet investigated their influence on prognosis in non-small cell lung cancer (NSCLC). We aimed to explore their prognostic significance in NSCLC, in addition to examining associations with previously investigated markers.

**Methods:**

Resected tumor tissue from 335 consecutive unselected stage I-IIIA NSCLC patients (1990–2005) were collected. Immunohistochemistry was used to evaluate the expression of CXCR6 and CXCL16 on tissue microarrays. *In vitro*, NSCLC cells (NCI-H460, A549 cells) were transfected with CXCL16 siRNA to examine effects on proliferation.

**Results:**

In univariate analysis, ↑ stromal cell CXCL16 expression was a significant positive prognostic factor (P = 0.016). CXCR6 was expressed in cancer cells, but did not show any prognostic impact. In the multivariate analysis, combined ↑cancer, and ↑stromal cell CXCL16 expression was an independent positive prognostic factor when compared to ↓stromal and ↓cancer cell expression (HR: 0.42; 95 % CI: 0.20–0.88; P = 0.022). Knockdown of CXCL16 by siRNA resulted in accelerated proliferation of NSCLC cell lines.

**Conclusion:**

We have shown that combined ↑cancer and ↑stromal cell CXCL16 expression is an independent positive prognostic factor in NSCLC. Further studies are warranted to elucidate the biological mechanism underlying this finding.

## Background

Lung cancer is the leading cause of cancer death worldwide [1]. Non-small cell lung cancer (NSCLC) is the predominant form of lung cancer, representing 80–85 % of new cases. Despite advances in treatment, NSCLC mortality remains high as the majority of patients present with advanced disease and are not candidates for curative surgery. The 5-year survival rates for surgically resected NSCLC range from 73 % to 24 % according to pathological stage [2], and many patients ultimately relapse and succumb to metastatic disease. New biological markers may improve outcome prediction and selection of additional therapy in NSCLC.

Chemokines are chemotactic cytokines originally recognized for their ability to induce leucocyte migration [3], are now known to be involved in a variety of pathologic and physiologic processes [4]. In cancer biology, chemokines are associated with tumor progression [5], metastasis [6] and angiogenesis [7], in addition to leukocyte recruitment to the tumor microenvironment [8]. Chemokines have been recognized as targets in cancer therapy as well as potential agents for immunotherapy, reflecting their multifaceted role in the development and progression of cancer [9, 10] .

The chemokine receptor CXCR6 was originally identified as a co-receptor for the human immunodeficiency virus (HIV) [11–13] and is expressed on subsets of CD4+ and CD8+ T-cells [14], plasma cells [15] and NK-cells [16]. Its ligand CXCL16, one of two chemokines known to exist in both transmembrane and soluble forms, facilitates the recruitment, and adhesion of CXCR6 expressing cells [17, 18] and is also a scavenger receptor for oxidized low-density lipoprotein [19]. CXCL16 is expressed on macrophages, dendritic cells, B-cells, and monocytes [17, 20], but is also constitutively expressed on epidermal keratinocytes [21], bronchial epithelial cells [22] and renal podocytes [23]. In addition to their roles in leucocyte recruitment and inflammation, CXCR6, and CXCL16 have been shown to influence angiogenesis [24, 25].

The expression of CXCL16 and CXCR6 has been investigated in a variety of human cancers [26] and correlated with both improved [27] and reduced survival [25]. An aptamer- found reduced expression of CXCL16 in NSCLC tissue compared to normal controls suggesting CXCL16 as a novel biomarker in NSCLC [28]. However, no studies have examined the impact by CXCR6 and CXCL16 on lung cancer survival. Hence, we examined the expression of CXCL16 and CXCR6 and their relations to prognosis in 335 unselected patients with NSCLC, and investigated possible relationships with our previously studied immunologic and angiogenic markers. Besides, the influence of CXCL16 on NSCLC cell proliferation was examined *in vitro*.

## Methods

### Patients

Patients surgically resected for stage I-IIIA NSCLC at the University Hospital of North Norway (UNN) and Nordland Hospital (NH) from 1990 through 2005 were included in this study. Of the 371 patients identified from the hospital databases, a total of 36 were excluded due to inadequate paraffin-embedded fixed tissue blocks (n = 13), other malignancy within 5 years prior to NSCLC diagnosis (n = 13), or radio-, or chemotherapy prior to surgery (n = 10). Thus, 335 patients were included in the study, 159 from UNN, and 176 from NH. Adjuvant chemotherapy had not been introduced in Norway during this period (1990–2004). This study includes follow up data as of January 2011. Patients were staged according to the revised 7th edition of UICC TNM classification of lung cancer [2]. The study was approved by The Norwegian Data Inspectorate and The Regional Committee for Medical and Health Research Ethics. Information about the study and subsequent written consent from patients was considered. However, as this was a retrospective study with more than half of patients deceased, with the rest of the patients having to be reminded about the death rate of the disease and the possible raising of unrealistic hope for the individual, The Norwegian Data Inspectorate, and The Regional Committee for Medical and Health Research Ethics waived the need for consent. All patient data were anonymized after collecting the clinicopathological variables for each patient and before doing the statistical analyses.

### Microarray construction

Two pathologists (S.Al-S and K.Al-S) reviewed the pathological specimens. Two representative areas of cancer cells (neoplastic epithelium) and two from the tumor surrounding stroma were marked on the paraffin donor blocks. Using a 0.6 mm-diameter stylet, one core from each marked area was transferred to a recipient block. Normal lung tissue localized distant from the tumor, in addition to samples from 20 patients without a diagnosis of cancer were used as controls. To include all tissue samples, a total of 9 TMAs were constructed. Multiple 4-μm-sections were cut with a Micron microtome (HM355S) before antibody staining for immunohistochemical analysis. The detailed methodology has been previously reported [29].

### Immunohistochemistry

**Fig. 1:**
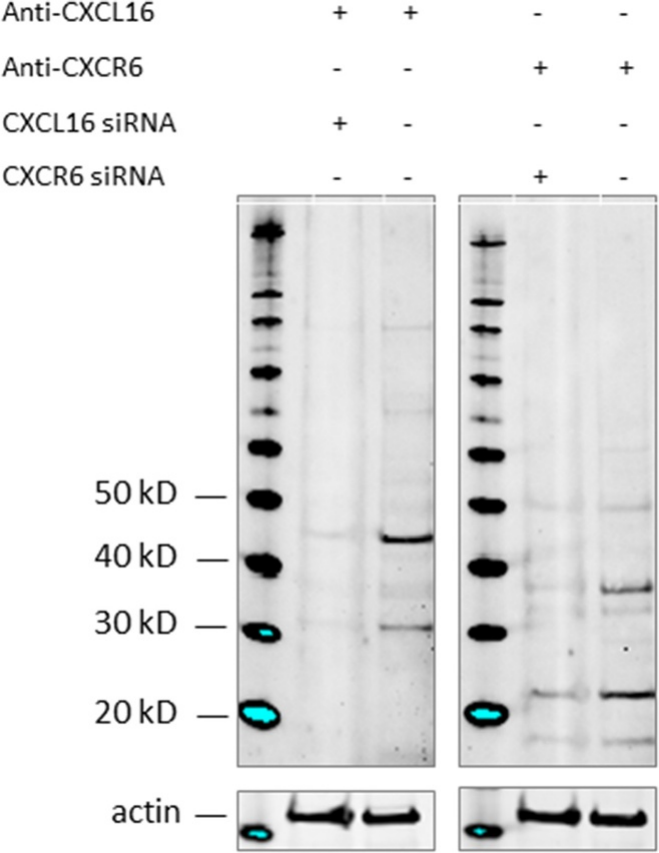
Antibody validation. In the lung adenocarcinoma cell line A549 we detected protein bands corresponding to CXCL16 and CXCR6. Transfection with specific siRNAs caused a marked decrease of protein expression. Equal loading was ensured by staining for beta-actin

CXCR6 (goat polyclonal, ab125115, 1:100), and CXCL16 (rabbit polyclonal, ab101404, 1:100) antibodies from Abcam were used in the study. The antibodies were validated by the manufacturer for immunohistochemistry (IHC) on paraffin-embedded material. In addition, in-house validation by Western blot analysis was performed (Fig. 1). Cut sections were deparaffinized with xylene and rehydrated with ethanol. Antigen retrieval was done by placing the sections in 0.01 M citrate buffer pH 6.0 before microwave heating for 20 minutes at 450 W. Endogenous peroxidase was blocked by incubation in 3 % H_2_0_2_ for 10 minutes. Sections were blocked in 5 % goat or rabbit serum for 1 hour before overnight incubation with the primary antibodies at 4 °C. The primary antibodies were visualized by adding a secondary biotin-conjugated antibody followed by an Avidin/Biodin/Peroxidase complex (Vectastain ABC Elite-kit, Vectastain), and substrate (Vector NovaRed, Vectastain). As negative staining controls, the primary antibodies were replaced with the primary antibody diluent. All slides were counterstained with haematoxylin to visualize the nuclei.

### Scoring of Immunohistochemistry

Sections were scored semi-quantitatively by two experienced pathologist (S.Al-S and E.R). The dominant staining intensity of stromal and cancer cells was scored as: 0 = negative, 1 = weak, 2 = intermediate, 3 = strong. For stromal CXCL16 and cancer cell CXCR6 density was also scored, in the following manner: 0 = no cells showing positivity, 1 = less than 25 % positivity, 2 = 25-50 % positivity, and 3 = 50-100 % positivity. CXCL16 displayed homogenous staining in cancer cells, while CXCR6 did not show positivity in stromal cells. For some patients, one, or both of the stromal or cancer tissue cores were missing. Favoring a conservative approach, we chose not to extrapolate scores from single cores. Consequently, all reported marker expressions are based on the evaluation of two separate tissue cores.

The mean intensity score of duplicate cores from each patient was calculated. For stromal CXCL16 and cancer cell CXCR6, this intensity score was combined with the mean density score. Finally, protein expression was dichotomized into high and low expression. High cancer cell CXCR6 was defined as a score **≥**5.5, while high cancer, and stromal cell CXCL16 was defined as scores ≥2.5 and ≥3, respectively. The stromal, and cancer cell CXCL16 co-expression variable was constructed by combining the dichotomized cancer and stromal cell CXCL16 variables.

### Western blotting

The specificity of the CXCL16 and CXCR6 antibodies was investigated by Western blotting. Cell lysates were incubated with NuPAGE LDS Sample Buffer (Life Technologies, USA) for 5 minutes at 85 °C, sonicated briefly, and run on a NuPAGE® 4-12 % Bis Tris Gel (cat#NP0322, Life Technologies, USA). Blotting was performed on a Hybond nitrocellulose membrane (cat# RPN2020D, GE Healthcare) using the NuPAGE blotting system (Life Technologies, USA). The membrane was incubated with Odyssey blocking buffer (cat# 927–40000, LI-COR Biosciences, Germany) for 1 hour at room temperature. Primary and secondary antibodies were diluted in the blocking buffer. Anti-CXCL16 antibody (cat# 101404) was used in the dilution of 1:500, anti-CXCR6 in the dilution 1:500 (cat# 125115) and anti-actin (cat#A2066, Sigma) 1:2000. IRDye CW secondary antibodies (cat# 926–32213 and 926–68073, LI-COR, Germany) were used in dilution 1:10000. Molecular weight markers used were SeeBlue Plus 2 (cat# LC5925, Life Technologies, USA), and Magic Mark XP (cat# LC5602, Life Technologies, USA). Images were acquired on the ODYSSEY Sa Infrared Imaging System (LI-COR, Germany).

### Cell cultures

NCI-H460 cells (ATCC#HTB-177) were grown in RPMI-1640 media (21875–034, Gibco), A549 cells (ATCC# CCL-185) were grown in Ham’s F-12 K (Kaighn’s) media (21127–022, Gibco). Media for all cell lines contained 10 % fetal calf serum (Biochrome, Berlin, Germany), and 1 % of mixed 100 U/ml penicillin and 100 mg/ml streptomycin (Sigma, cat#P0781, Sigma-Aldrich, St. Louis, MO).

### RNA interference

Cells were transfected either in 6-well plates (for antibody specificity validation) or in E-plates 16 (Roche, cat# 05469830001) with human CXCL16 Silencer Select siRNA (Ambion, s33807), or CXCR6 Silencer Select siRNA (Ambion, s20963) using Lipofectamine 2000 (Invitrogen, cat# 11668–027, Carlsbad, CA) according to the manufacturer’s recommendations. A scrambled negative control siRNA was included in all experiments (Silencer Negative Control #2 siRNA, Ambion, Austin, TX). BLOCK-iT™ Fluorescent Oligo Reagent (cat# 2013, Invitrogen) was used for transfection efficiency control. Cells were harvested 24 hours after siRNA transfection.

### Proliferation assay

NCI-H460 and A549 cells were trypsinized briefly until detached. Cells were resuspended in complete growth media and counted. Optimal cell number per well (5000) was determined in initial titration experiments. According to xCELLigence (Roche) manufacturer’s instructions, cells were seeded in duplicates into E-plates 16 (Roche, cat# 05469830001) after baseline measurement. Plates were incubated for 1 hour at room temperature, and then placed into the RTCA DP instrument (Roche, cat# 05469759001) located in an incubator preserving same temperature and CO_2_ concentration as were used for routine cultivation of the cells. siRNA transfection mix was added to the cells 6 hours after seeding, and left there for 4 hours. After that, transfection mix was replaced with regular growth media. Cell index (arbitrary unit reflecting the cell-sensor impedance) was measured every 15 minutes during the first 4 hours for better resolution at attachment and spreading phase. Further measurements were taken every 30 minutes. Doubling times were calculated with RTCA software 1.2 (Roche). At least three independent experiments were performed.

### Statistical Methods

The Kaplan-Meier method was used to analyze the association between marker expression and disease-specific survival (DSS), which was determined from the date of surgery to the time of lung cancer death. The statistical significance of differences between survival curves was assessed with the log-rank test. Correlation assessments between marker expression and other variables was done using Spearman’s correlation test. Only variables with significant P-values from the univariate analyses were entered into the multivariate analysis, using the Cox proportional hazards model (backward stepwise, probability for stepwise entry, and removal set at 0.05 and 0.10). Statistical significance of the cell proliferation assays was determined using a two-sided Student’s *t*-test. P-values < 0.05 were considered statistically significant.

## Results

### Western blot

We used Western blotting to verify the specificity of the CXCL16 and CXCR6 antibodies (Fig. 1) as described. The molecular weight of the detected protein (strongest bands) corresponded well with the predicted weight and data provided by the manufacturers. The weaker extra bands may represent chemically modified or degraded proteins, or products of alternative splicing. siRNAs targeted against CXCL16 and CXCR6 caused marked decrease in the intensity of the bands, compared to scrambled control siRNA. This confirms the specificity of the used antibodies.

### Patient characteristics

The demographic and clinical characteristics of the patients are presented in Table 1. The median patient age was 67 (range 28–85) and the majority were male (76 %). Nearly all patients (96 %) were present or previous smokers. The median follow-up was 105 months (range 73 – 234). There were 191 squamous cell carcinomas (SCC), 113 adenocarcinomas (AC) and 31 large-cell carcinomas (LCC).

**Table 1:** Prognostic clinicopathologic variables as predictors of disease-specific survival in 335 NSCLC-patients

Characteristics	Patients N, (%)	Median survival (months)	5-year survival (%)	P
Age				.421
≤65 years	156 (47)	98	56	
>65 years	179 (53)	NR	60	
Sex				.220
Female	82 (24)	190	64	
Male	253 (76)	98	56	
Smoking status				.257
Never	15 (5)	19	43	
Previous	105 (31)	84	55	
Present	215 (64)	NR	60	
Performance status				.016
0	197 (59)	NR	63	
1	120 (36)	64	52	
2	18 (5)	25	33	
Weight loss				.759
<10 %	303 (90)	190	58	
>10 %	32 (10)	98	57	
Histology				.028
Squamous cell carcinoma	191 (57)	NR	66	
Adenocarcinoma	113 (34)	54	46	
Large cell carcinoma	31 (9)	98	56	
Differentiation				<.001
Poor	138 (41)	47	47	
Moderate	144 (43)	190	65	
Well	53 (16)	NR	68	
Surgical procedure				0.007
Wedge + Lobectomy	243 (73)	190	62	
Pneumectomy	92 (27)	37	47	
Pathological stage				<.001
pI	157 (47)	NR	72	
pII	136 (41)	62	51	
pIIIA	42 (12)	17	24	
Tumor stage				<.001
1	85 (25)	190	75	
2	188 (56)	84	57	
3	62 (19)	25	37	
Nodal stage				<.001
0	232 (69)	NR	67	
1	76 (23)	35	43	
2	27 (8)	18	18	
Surgical margins				.374
Free	307 (92)	190	59	
Not free	28 (8)	47	48	
Vascular infiltration				<.001
No	284 (85)	190	62	
Yes	51 (15)	27	33	

NR, not reached; NCSLC, non-small cell lung cancer

### Marker expression and correlations

**Fig. 2:**
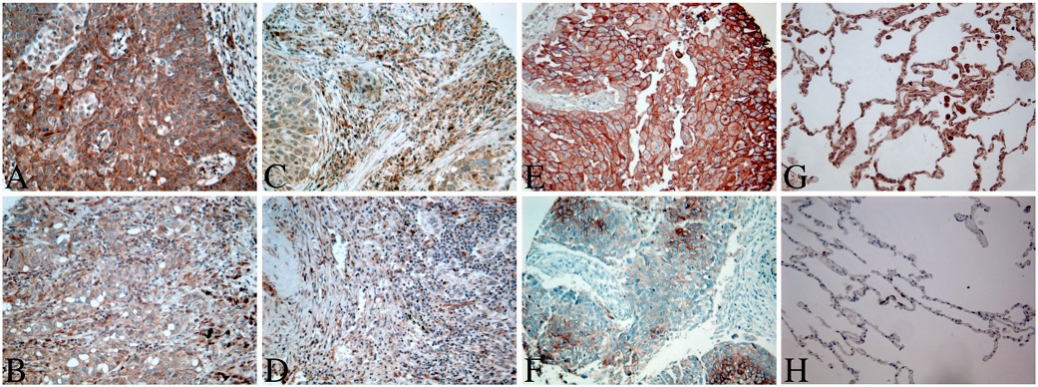
Immunohistochemical analyses of cancer and stromal cell CXCL16 expression and cancer cell CXCR6 in NSCLC. (**a**) Cancer cell CXCL16 high expression; (**b**) Cancer cell CXCL16 low expression; (**c**) stromal CXCL16 high expression; (**d**) stromal CXCL16 low expression; (**e**) Cancer cell CXCR6 high expression; (**f**) Cancer cell CXCR6 low expression; (**g**) Normal lung CXCL16 expression; (**h**) Normal lung CXCR6 expression

The expression of CXCL16 was predominantly cytoplasmic, while CXCR6 exhibited membranous staining (Fig. 2). CXCL16 was expressed in both stromal and cancer cells, whereas CXCR6 was only expressed in cancer cells (table 2). The stromal cells displaying positivity for CXCL16 were fibroblasts, endothelial cells, macrophages, and plasma cells. Of the controls (20 patients without a diagnosis of cancer), 50 % showed varying degrees of positivity for CXCR6 while 100 % showed strong positivity for CXCL16. There were no significant correlations between CXCR6 or CXCL16 and adaptive immunological (CD4, CD8, CD20), innate immunological (CD68, CD56, CD1A), or angiogenic (vascular endothelial growth factors and receptors, platelet derived growth factors, and receptors and fibroblast growth factor-2 and receptor-1) markers (data not shown). For both CXCR6 and CXCL16 the correlations with clinicopathological factors were only weak or not significant (r < 0.2). A correlation was observed between stromal and cancer cell CXCL16 expression (r = 0.368, P < 0.01), however no significant correlation was observed between CXCR6 and CXCL16 in cancer cells.

**Table 2:** CXCL16 and CXCR6 expression as predictors of disease-specific survival in 335 NSCLC patients

Characteristics	Patients, N (%)	Median survival (months)	5-year survival (%)	P
CXCL16				
Cancer cells				.080
High	48 (14)	NR	67	
Low	227 (68)	98	56	
Missing	60 (18)			
Stromal cells				.016
High	258 (77)	57	48	
Low	43 (13)	189	62	
Missing	34 (10)			
CXCR6				
Cancer cells				.093
High	41 (12)	190	73	
Low	245 (73)	122	55	
Missing	49 (15)			
CXCL16				
Cancer cells +				.016
stromal cells combined				
High/High	43 (13)	NR	71	
High/Low	182 (54)	190	58	
Low/Low	41 (12)	47	48	
Missing	69 (21)			

NR, not reached; NCSLC, non-small cell lung cancer

### Univariate analysis

The prognostic impacts of clinicopathologic variables on DSS are given in Table 1. WHO Performance status (P = 0.016), histology (P = 0.028), differentiation (P < 0.001), surgical procedure (P = 0.007), pathological stage (P < 0.001), tumor stage (P < 0.001), nodal stage (P < 0.001) and vascular infiltration (P <0.001) were significant prognosticators.

**Fig. 3:**
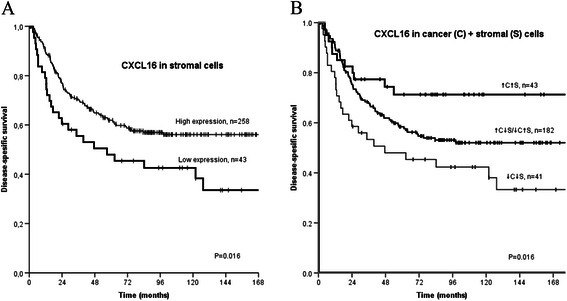
**a**. Disease specific survival curve according to expression of stromal CXCL16 in 301 NSCLC patients. **b**. Disease specific survival curve according to the co-expression of stromal and cancer cell CXCL16 in 266 NSCLC patients

The influence of marker expression on survival is presented in Table 2. High CXCL16 expression in stromal cells was significantly associated with an improved DSS (P = 0.016, Fig. 3a), with similar trends observed for each hospital separately (NH, P = 0.045; UNN, P = 0.137). The combination of high stromal and high cancer cell CXCL16 was also significantly associated with an improved DSS (P = 0.016, Fig. 3b). Cancer cell CXCR6 and CXCL16 did not have significant impact on survival in univariate analyses.

### Multivariate analysis

Significant clinicopathologic variables were entered into multivariate analysis in two separate models, one with stromal CXCL16 expression variable (model 1) and one with the co-expression variable of cancer and stromal cell CXCL16 (model 2).

Tumor stage, nodal stage, tumor differentiation, performance status, vascular infiltration, and histology were independent prognostic variables in both models (Table 3).

**Table 3:** Results of Cox regression analyses for clinicopathological factors and CXCL16 in stromal cells (model 1) and co-expression of CXCL16 in cancer and stromal cells (model 2*)

Factor	HR	CI 95 %	P
Tumor stage			<0.001^†^
T1	1.00		
T2	1.60	(0.97–2.64)	0.065
T3	3.80	(2.13–6.77)	<0.001
Nodal stage			<0.001^†^
N0	1.00		
N1	2.01	(1.30–3.11)	0.002
N2	3.08	(1.74–5.44)	<0.001
Differentiation			0.009^†^
Well	1.00		
Poor	1.17	(1.12–3.85)	0.020
Moderate	2.08	(0.63–2.20)	0.618
Performance status			0.040^†^
0	1.00		
1	1.52	(1.02–2.26)	0.040
2	2.15	(0.94–4.91)	0.069
Vascular infiltration			
No	1.00		
Yes	1.70	(1.01–2.87)	0.046
Histology			<0.001^†^
Squamous carcinoma	1.00		
Adenocarcinoma	2.23	(1.48–3.35)	<0.001
Large cell carcinoma	0.80	(0.39–1.66)	0.555
CXC16 stromal cells			
Low	1		
High	0.55	(0.35–0.87)	0.011
CXCL16 cancer and stromal cells*			0.031^†^
Low/Low	1.00		
Low/High + High/Low	0.57	(0.35–0.93)	0.023
Low/Low	0.42	(0.20–0.88)	0.022

^†^Overall significance as prognostic marker. HR, hazard ratio; CI 95 %, 95 % confidence interval

In model 1, high expression of stromal CXCL16 was an independent positive prognostic factor (HR: 0.55; 95 % CI: 0.35 – 0.87, P = 0.011). The combined high expression of CXCL16 in stromal and cancer cells was an independent positive prognostic factor for DSS in model 2 (HR: 0.42; 95 % CI: 0.20 – 0.88, P = 0.022) when compared to the combined low expression.

### Cell proliferation

**Fig. 4:**
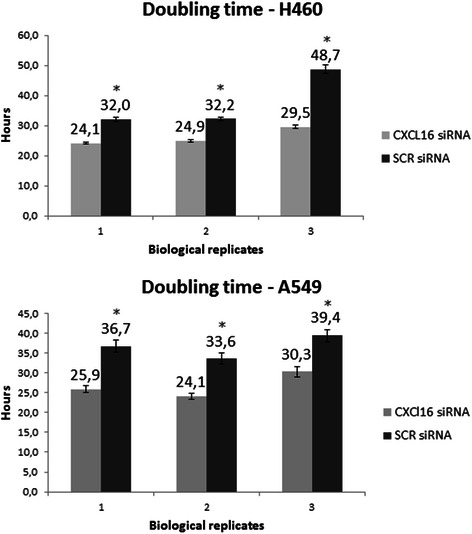
Knockdown of CXCL16 with siRNA caused activation of proliferation compared to the negative scrambled control in NSCLC cell lines A549 and NCI-H460. Cells were trypsinized briefly until detached, resuspended in complete growth media and counted. According to the manufacturer’s instructions, cells were seeded in duplicates into E-plates 16 after baseline measurement. Plates were incubated for 1 hour at room temperature, and then placed into the RTCA DP instrument located in an incubator preserving same temperature and CO_2_ concentration as were used for routine cultivation of the cells. siRNA transfection mix was added to the cells 6 hours after seeding, and left there for 4 hours. Subsequently, the transfection mix was replaced with regular growth media. Cell index (arbitrary unit reflecting the cell-sensor impedance) was measured every 15 minutes during the first 4 hours for better resolution at attachment and spreading phase. Further measurements were taken every 30 minutes. Doubling times were calculated with RTCA software 1.2 (Roche). ± S.D of 4 technical replicates are shown. * P <0.001

The increased survival seen in patients with combined high CXCL16 expression in cancer and stromal cells led us to investigate the effect of CXCL16 on cell proliferation. We employed the xCELLigence platform (Roche) which facilitates studying of cell proliferation in real time. This is a micro electric assay based on changing impedance of bottom electrodes in presence of the cells. Attachment and initial spreading of the cells typically took 3–6 hours, after which cells were transfected with siRNAs. We repeatedly observed that knockdown of CXCL16 with siRNA caused activation of proliferation compared to the negative scrambled control (P < 0.001, Fig. 4). This was evident both from the growth curves and from the doubling time calculations. The same effect was observed in two different NSCLC cell lines: A549 and NCI-H460.

## Discussion

We present the first study on the prognostic impact of the chemokines CXCL16 and CXCR6 in lung cancer. To our knowledge, this is also the first study examining their expression in the tumor surrounding stroma in addition to the epithelial cancer cells. Utilizing TMA methodology on an unselected NSCLC cohort from two hospitals, we show that high stromal CXCL16 expression as well as combined high stromal/cancer cell expression of CXCL16 is associated with a favorable DSS. The patterns of increased, intermediate, and reduced CXCL16 expression showed 5-year survival rates of 71 %, 58 % and 48 %, respectively. Supporting our findings, a siRNA-mediated knockdown of CXCL16 resulted in accelerated cell proliferation in two different NSCLC cell lines.

The impact of CXCL16 and CXCR6 expression on survival varies markedly between different malignancies. In colorectal cancer, Hojo et al. showed that high levels of CXCL16 expression in tumors was a positive prognostic factor and was correlated with increased levels of tumor-infiltrating lymphocytes [30]. Similarly, radiation induced CXCL16 expression stimulated the recruitment of antitumor CD8 + cells in a murine model of breast cancer [31]. We have previously shown that infiltrating lymphocytes are strongly associated with improved survival in NSCLC [32, 33], but we found no correlation between markers of tumor-infiltrating lymphocytes and CXCL16 levels in cancer cells or tumor stroma. If CXCL16 plays a role in leukocyte recruitment in NSCLC, this is not reflected by corresponding levels of immune cell markers in the resected tissue. In gastric cancer, Xing et al. found nuclear CXCL16 expression to be related to improved survival and reduced cancer aggressiveness [34]. We did, however, not observe nuclear staining for CXCL16 in our TMAs.

Gutwein et al. studied CXCL16 and CXCR6 expression in renal cancer, and reported low CXCL16 expression to be linked to decreased overall survival [27]. Interestingly, both normal lung [22] and renal [23] tissue show high endogenous CXCL16 expression, which may suggest that reduced or aberrant CXCL16 expression is linked to cancer development in these organs. That loss of endogenous CXCL16 expression is a factor in the development of NSCLC is further supported by the study conducted by Mehan et al., which showed reduced CXCL16 protein levels in NSCLC tissue compared to normal tissue [28]. Moreover, the increased proliferation we observed when CXCL16 was knocked down using siRNA suggest a negative influence of CXCL16 on NSCLC development. CXCL16, being a transmembrane chemokine, has previously been shown to be involved in cell adhesion through binding to CXCR6 [18, 35]. Loss of cell adhesion may therefore contribute to explaining why CXCL16 knockdown leads to increased cell proliferation through the loss of contact inhibition. Decreased cell adhesion upon loss of CXCL16 expression needs yet to be experimentally confirmed, but if it indeed takes place, it might promote detachment of single cancer cells, their migration, and establishment elsewhere as metastatic foci, thus contributing to worse prognosis.

CXCL16 and CXCR6 are up-regulated in many cancers, and increased expression has been linked to more aggressive disease and advanced tumor stage [25, 36, 37], though few studies have shown increased expression to be independently linked to reduced survival. A recent study of CXCR6 in resected hepatocellular carcinoma found high CXCR6 expression to be associated with reduced survival in a multivariate model, through stimulation of a pro-inflammatory tumor microenvironment [25]. In contrast, loss of CXCR6 expression on NKT-cells resulted in increased liver metastasis in a murine model [38]. Our results do not indicate a prognostic role for CXCR6 in NSCLC, though it was highly expressed in many NSCLC tissue cores.

We did not observe CXCR6 expression on cells in the tumor stroma. CXCR6 has previously been reported to be involved in T cell recruitment to the lung [39], nevertheless no staining for CXCR6 was seen on infiltrating lymphocytes in our TMAs. While expression has been reported on T-cells in inflammatory lung disease such as sarcoidosis [40], our result indicate that CXCR6 is not expressed to an appreciable degree in infiltrating immune cells in NSCLC.

Chemokines containing the ELR motif (ELR+) are known to promote angiogenesis [41]. Despite being ELR negative, CXCL16 has been shown to be an angiogenic factor for recruitment of human umbilical vein endothelial cells [24] and an important mediator of angiogenesis in rheumatoid arthritis [42]. In prostate cancer cell lines, CXCL16 induced the expression of progangiogenic cytokines IL-8 and VEGF, while CXCR6 expression on implanted tumor cells lead to increased blood vessel formation in mice [36]. Similarly, knockdown of CXCR6 leads to reduced angiogenesis in murine hepatocellular carcinoma [25]. However, we did not observe a significant association between CXCR6 and CXCL16 expression and markers of angiogenesis in NSCLC.

The extracellular domain of CXCL16 can be proteolytically cleaved by the disintegrin-like metalloproteinase ADAM10, yielding a soluble form (sCXCL16) [43]. Hu et al. reported that sCXCL16 increased the invasive capacity of NSCLC cells, which suggests that CXCL16 may have different roles in lung cancer depending on whether it exists in a soluble or cellular form [44]. In a recent study in ovarian cancer, high serum sCXCL16 was linked to poor prognosis [45], whereas tumor CXCL16 and CXCR6 were not associated with survival. In colorectal cancer, high tumor CXCL16 expression has been correlated with a favorable prognosis [30], while high sCXCL16 is linked to recurrence [46]. The relationship between serum sCXCL16 and tissue CXCL16 remains unknown in NSCLC, and should be scrutinized in future studies.

## Conclucions

We have shown that high stromal cell CXCL16 expression and combined high stromal and cancer cell CXCL16 are independent positive prognostic factors in NSCLC. Our finding is supported by the fact that knockdown of CXCL16 results in increased cell proliferation in NSCLC cell lines.

## References

[1] Siegel R, Naishadham D, Jemal A (2013). Cancer statistics. CA Cancer J Clin.

[2] Rami-Porta R, Crowley JJ, Goldstraw P (2009). The revised TNM staging system for lung cancer. Ann Thorac Cardiovasc Surg.

[3] Baggiolini M (1998). Chemokines and leukocyte traffic. Nature.

[4] Raman D, Sobolik-Delmaire T, Richmond A (2011). Chemokines in health and disease. Exp Cell Res.

[5] Raman D, Baugher PJ, Thu YM, Richmond A (2007). Role of chemokines in tumor growth. Cancer Lett.

[6] Muller A, Homey B, Soto H, Ge N, Catron D, Buchanan ME (2001). Involvement of chemokine receptors in breast cancer metastasis. Nature.

[7] Dimberg A. Chemokines in angiogenesis. Current topics in microbiology and immunology 2010;341:59–80. 10.1007/82_2010_21 20373091

[8] Balkwill F (2004). Cancer and the chemokine network. Nat Rev Cancer.

[9] Homey B, Muller A, Zlotnik A (2002). Chemokines: agents for the immunotherapy of cancer?. Nat Rev Immunol.

[10] Lazennec G, Richmond A (2010). Chemokines and chemokine receptors: new insights into cancer-related inflammation. Trends Mol Med.

[11] Liao F, Alkhatib G, Peden KW, Sharma G, Berger EA, Farber JM (1997). STRL33, A novel chemokine receptor-like protein, functions as a fusion cofactor for both macrophage-tropic and T cell line-tropic HIV-1. J Exp Med.

[12] Loetscher M, Amara A, Oberlin E, Brass N, Legler D, Loetscher P (1997). TYMSTR, a putative chemokine receptor selectively expressed in activated T cells, exhibits HIV-1 coreceptor function. CB.

[13] Deng HK, Unutmaz D, KewalRamani VN, Littman DR (1997). Expression cloning of new receptors used by simian and human immunodeficiency viruses. Nature.

[14] Kim CH, Kunkel EJ, Boisvert J, Johnston B, Campbell JJ, Genovese MC (2001). Bonzo/CXCR6 expression defines type 1-polarized T-cell subsets with extralymphoid tissue homing potential. J Clin Invest.

[15] Nakayama T, Hieshima K, Izawa D, Tatsumi Y, Kanamaru A, Yoshie O (2003). Cutting Edge: Profile of Chemokine Receptor Expression on Human Plasma Cells Accounts for Their Efficient Recruitment to Target Tissues. J Immunol.

[16] Paust S, Gill HS, Wang BZ, Flynn MP, Moseman EA, Senman B (2010). Critical role for the chemokine receptor CXCR6 in NK cell-mediated antigen-specific memory of haptens and viruses. Nat Immunol.

[17] Matloubian M, David A, Engel S, Ryan JE, Cyster JG (2000). A transmembrane CXC chemokine is a ligand for HIV-coreceptor Bonzo. Nat Immunol.

[18] Shimaoka T, Nakayama T, Fukumoto N, Kume N, Takahashi S, Yamaguchi J (2004). Cell surface-anchored SR-PSOX/CXC chemokine ligand 16 mediates firm adhesion of CXC chemokine receptor 6-expressing cells. J Leukoc Biol.

[19] Shimaoka T, Kume N, Minami M, Hayashida K, Kataoka H, Kita T (2000). Molecular Cloning of a Novel Scavenger Receptor for Oxidized Low Density Lipoprotein, SR-PSOX, on Macrophages. J Biol Chem.

[20] Wilbanks A, Zondlo SC, Murphy K, Mak S, Soler D, Langdon P (2001). Expression Cloning of the STRL33/BONZO/TYMSTR Ligand Reveals Elements of CC, CXC, and CX3C Chemokines. J Immunol.

[21] Scholz F, Schulte A, Adamski F, Hundhausen C, Mittag J, Schwarz A (2007). Constitutive expression and regulated release of the transmembrane chemokine CXCL16 in human and murine skin. J Invest Dermatol.

[22] Day C, Patel R, Guillen C, Wardlaw AJ (2009). The chemokine CXCL16 is highly and constitutively expressed by human bronchial epithelial cells. Exp Lung Res.

[23] Schramme A, Abdel-Bakky MS, Gutwein P, Obermuller N, Baer PC, Hauser IA (2008). Characterization of CXCL16 and ADAM10 in the normal and transplanted kidney. Kidney Int.

[24] Zhuge X, Murayama T, Arai H, Yamauchi R, Tanaka M, Shimaoka T (2005). CXCL16 is a novel angiogenic factor for human umbilical vein endothelial cells. Biochem Biophys Res Commun.

[25] Gao Q, Zhao YJ, Wang XY, Qiu SJ, Shi YH, Sun J (2012). CXCR6 upregulation contributes to a proinflammatory tumor microenvironment that drives metastasis and poor patient outcomes in hepatocellular carcinoma. Cancer Res.

[26] Deng L, Chen N, Li Y, Zheng H, Lei Q (1806). CXCR6/CXCL16 functions as a regulator in metastasis and progression of cancer. Biochim Biophys Acta.

[27] Gutwein P, Schramme A, Sinke N, Abdel-Bakky MS, Voss B, Obermuller N (2009). Tumoural CXCL16 expression is a novel prognostic marker of longer survival times in renal cell cancer patients. Eur J Cancer.

[28] Mehan MR, Ayers D, Thirstrup D, Xiong W, Ostroff RM, Brody EN (2012). Protein Signature of Lung Cancer Tissues. PLoS One.

[29] Bremnes RM, Veve R, Gabrielson E, Hirsch FR, Baron A, Bemis L (2002). High-throughput tissue microarray analysis used to evaluate biology and prognostic significance of the e-cadherin pathway in non–small-cell lung cancer. J Clin Oncol.

[30] Hojo S, Koizumi K, Tsuneyama K, Arita Y, Cui Z, Shinohara K (2007). High-level expression of chemokine CXCL16 by tumor cells correlates with a good prognosis and increased tumor-infiltrating lymphocytes in colorectal cancer. Cancer Res.

[31] Matsumura S, Wang B, Kawashima N, Braunstein S, Badura M, Cameron TO (2008). Radiation-induced CXCL16 release by breast cancer cells attracts effector t cells. J Immunol.

[32] Al-Shibli KI, Donnem T, Al-Saad S, Persson M, Bremnes RM, Busund LT (2008). Prognostic effect of epithelial and stromal lymphocyte infiltration in non-small cell lung cancer. Clin Cancer Res.

[33] Hald SM, Bremnes RM, Al-Shibli K, Al-Saad S, Andersen S, Stenvold H (2013). CD4/CD8 co-expression shows independent prognostic impact in resected non-small cell lung cancer patients treated with adjuvant radiotherapy. Lung Cancer.

[34] Xing Y-n, Xu X-y, Nie X-c, Yang X, Yu M, Xu H-m (2012). Role and clinicopathologic significance of CXC chemokine ligand 16 and chemokine (C-X-C motif) receptor 6 expression in gastric carcinomas. Hum Pathol.

[35] Chandrasekar B, Bysani S, Mummidi S (2004). CXCL16 signals via gi, phosphatidylinositol 3-kinase, Akt, IκB kinase, and nuclear factor-κb and induces cell-cell adhesion and aortic smooth muscle cell proliferation. J Biol Chem.

[36] Wang J, Lu Y, Wang J, Koch AE, Zhang J, Taichman RS (2008). CXCR6 induces prostate cancer progression by the AKT/mammalian target of rapamycin signaling pathway. Cancer Res.

[37] Wente MN, Gaida MM, Mayer C, Michalski CW, Haag N, Giese T (2008). Expression and potential function of the CXC chemokine CXCL16 in pancreatic ductal adenocarcinoma. Int J Oncol.

[38] Cullen R, Germanov E, Shimaoka T, Johnston B (2009). Enhanced tumor metastasis in response to blockade of the chemokine receptor cxcr6 is overcome by nkt cell activation. J Immunol.

[39] Morgan AJ, Guillen C, Symon FA, Huynh TT, Berry MA, Entwisle JJ (2005). Expression of CXCR6 and its ligand CXCL16 in the lung in health and disease. Clin Exp Allergy.

[40] Agostini C, Cabrelle A, Calabrese F, Bortoli M, Scquizzato E, Carraro S (2005). Role for CXCR6 and its ligand CXCL16 in the pathogenesis of T-cell alveolitis in sarcoidosis. Am J Respir Crit Care Med.

[41] Strieter RM, Burdick MD, Gomperts BN, Belperio JA, Keane MP (2005). CXC chemokines in angiogenesis. Cytokine & Growth Factor Rev.

[42] Isozaki T, Arbab AS, Haas CS, Amin MA, Arendt MD, Koch AE (2013). Evidence that cxcl16 is a potent mediator of angiogenesis and is involved in endothelial progenitor cell chemotaxis : studies in mice with k/bxn serum-induced arthritis. Arthritis Rheum.

[43] Gough PJ, Garton KJ, Wille PT, Rychlewski M, Dempsey PJ, Raines EW (2004). A disintegrin and metalloproteinase 10-mediated cleavage and shedding regulates the cell surface expression of cxc chemokine ligand 16. J Immunol.

[44] Hu W, Liu Y, Zhou W, Si L, Ren L (2014). CXCL16 and CXCR6 are coexpressed in human lung cancer in vivo and mediate the invasion of lung cancer cell lines in vitro. PLoS One.

[45] Gooden MJ, Wiersma VR, Boerma A, Leffers N, Boezen HM, Ten Hoor KA (2014). Elevated serum CXCL16 is an independent predictor of poor survival in ovarian cancer and may reflect pro-metastatic ADAM protease activity. Br J Cancer.

[46] Matsushita K, Toiyama Y, Tanaka K, Saigusa S, Hiro J, Uchida K (2012). Soluble CXCL16 in preoperative serum is a novel prognostic marker and predicts recurrence of liver metastases in colorectal cancer patients. Ann Surg Oncol.

