# Erratum to: Prognostic impact of CXCL16 and CXCR6 in non-small cell lung cancer: combined high CXCL16 expression in tumor stroma and cancer cells yields improved survival

**DOI:** 10.1186/s12885-016-2954-1

**Published:** 2016-11-24

**Authors:** Sigurd M. Hald, Yury Kiselev, Samer Al-Saad, Elin Richardsen, Charles Johannessen, Marte Eilertsen, Thomas K. Kilvaer, Khalid Al-Shibli, Sigve Andersen, Lill-Tove Busund, Roy M. Bremnes, Tom Donnem

**Affiliations:** 1 Department of Clinical Medicine, UiT The Arctic University of Norway, 9037 Tromso, Norway; 2 Department of Oncology, University Hospital of North Norway, Tromso, Norway; 3 Department of Medical Biology, UiT The Arctic University of Norway, Tromso, Norway; 4 Department of Pathology, Nordland Hospital, Bodo, Norway; 5 Department of Clinical Pathology, UniversityHospital of North Norway, Tromso, Norway; 6 Department of Pharmacy, UiT The Arctic University of Norway, Tromso, Norway

## Erratum

After publication of the original article [1], the author noticed that there are some error with Table 2 and Table 3. Please see the correct Tables below. We apologize for any inconvenience caused.

**Table 2:** CXCL16 and CXCR6 expression as predictors of disease-specific survival in 335 NSCLC patients

Characteristics	Patients, N (%)	Median survival (months)	5-year survival (%)	P
CXCL16				
Cancer cells				.080
High	48 (14)	NR	67	
Low	227 (68)	98	56	
Missing	60 (18)			
Stromal cells				**.016**
High	258 (77)	189	62	
Low	43 (13)	57	48	
Missing	34 (10)			
CXCR6				
Cancer cells				.093
High	41 (12)	190	73	
Low	245 (73)	122	55	
Missing	49 (15)			
CXCL16				
Cancer cells + stromal cells combined				**.016**
High/High	43 (13)	NR	71	
High/Low	182 (54)	190	58	
Low/Low	41 (12)	47	48	
Missing	69 (21)			

NR, not reached; NCSLC, non-small cell lung cancer. Bold values indicate p < 0.05

**Table 3:** Results of multivariate Cox regression analyses for clinicopathological factors and CXCL16 in stromal cells (model 1) and co-expression of CXCL16 in cancer and stromal cells (model 2*)

Factor	HR	CI 95%	P
T-status			**<0.001** ^**†**^
T1	1.00		
T2	1.60	(0.97-2.64)	0.065
T3	3.80	(2.13-6.77)	**<0.001**
N-status			**<0.001** ^**†**^
N0	1.00		
N1	2.01	(1.30-3.11)	**0.002**
N2	3.08	(1.74-5.44)	**<0.001**
Differentiation			**0.009** ^**†**^
Well	1.00		
Moderate	1.17	(0.63-2.20)	0.618
Poor	2.08	(1.12-3.85)	**0.020**
Performance status			**0.040** ^**†**^
ECOG 0	1.00		
ECOG 1	1.52	(1.02-2.26)	**0.040**
ECOG 2	2.15	(0.94-4.91)	0.069
Vascular infiltration			
No	1.00		
Yes	1.70	(1.01-2.87)	**0.046**
Histology			**<0.001** ^**†**^
Squamous carcinoma	1.00		
Adenocarcinoma	2.23	(1.48-3.35)	**<0.001**
Large cell carcinoma	0.80	(0.39-1.66)	0.555
CXC16 stromal cells			
Low	1		
High	0.55	(0.35-0.87)	**0.011**
CXCL16 cancer and stromal cells*			**0.031** ^**†**^
Low/Low	1.00		
Low/High + High/Low	0.57	(0.35-0.93)	**0.023**
High/High	0.42	(0.20-0.88)	**0.022**

^†^Overall significance as prognostic marker. HR, Hazard ratio. Bold values indicate p < 0.05
